# Topical sirolimus for the treatment of verrucous epidermal nevus associated with an *HRAS* mosaic mutation: a case report

**DOI:** 10.3389/fmed.2026.1932228

**Published:** 2026-08-24

**Authors:** Qiu-ju Wei, Wan-yan Xiang, Wen-jun Zheng, Qiu-ju Li

**Affiliations:** Department of Dermatology, The First Affiliated Hospital of Guangxi Medical University, Nanning, China

**Keywords:** *HRAS*, mosaic mutation, mTOR inhibitor, topical sirolimus, verrucous epidermal nevus

## Abstract

**Introduction:**

Verrucous epidermal nevus is a benign epidermal hamartoma associated with somatic mosaic mutations, which shows poor response to conventional therapies and has a high risk of recurrence. To date, there are no standardized treatment guidelines for this condition. In recent years, targeted treatments for specific gene mutations have provided new directions for the clinical management of this disease.

**Case report:**

We report a case of a 13-year-old child with verrucous epidermal nevus in whom genetic testing revealed an *HRAS* mosaic mutation. The child showed poor response to conventional treatments, including surgical excision, topical retinoic acid cream, and oral acitretin. However, after treatment with topical 0.5% (w/w) sirolimus ointment, the child’s skin lesions showed reduced area, thinning, improved pigmentation, and decreased roughness, and no local or systemic adverse reactions were observed during the 6-month treatment period.

**Conclusion:**

This case suggests that topical sirolimus may represent a potentially effective therapeutic option for patients with verrucous epidermal nevus who are refractory to conventional therapy.

## Introduction

Verrucous epidermal nevus (VEN) is a benign epidermal hamartoma, clinically presenting as well-defined, verrucous papules or plaques ranging from skin-colored to dark brown (1). The disease typically manifests at birth or during infancy (2). Its pathogenesis is associated with somatic mosaic mutations during embryonic development, but the specific molecular biological mechanism remains under investigation (3).

The treatment of VEN is hindered by multiple challenges, including the limited efficacy of conventional modalities such as topical medications, cryotherapy, electrocautery, and chemical peels, coupled with a high recurrence rate and the lack of established therapeutic protocols (4). However, with the in-depth study of the molecular pathogenesis of VEN, especially the discovery of driver gene mutations, targeted treatment has gradually become a new strategy in the clinical management of this disease. Sirolimus, as a mammalian target of rapamycin (mTOR) inhibitor, has been successfully used to treat proliferative skin lesions such as facial angiofibromas associated with tuberous sclerosis complex (TSC) (5), but its application in VEN still lacks systematic clinical reporting. This article reports a case of a child with an *HRAS* mosaic mutation-associated VEN who showed improvement in skin lesions after topical sirolimus treatment.

## Case presentation

**Figure 1:**
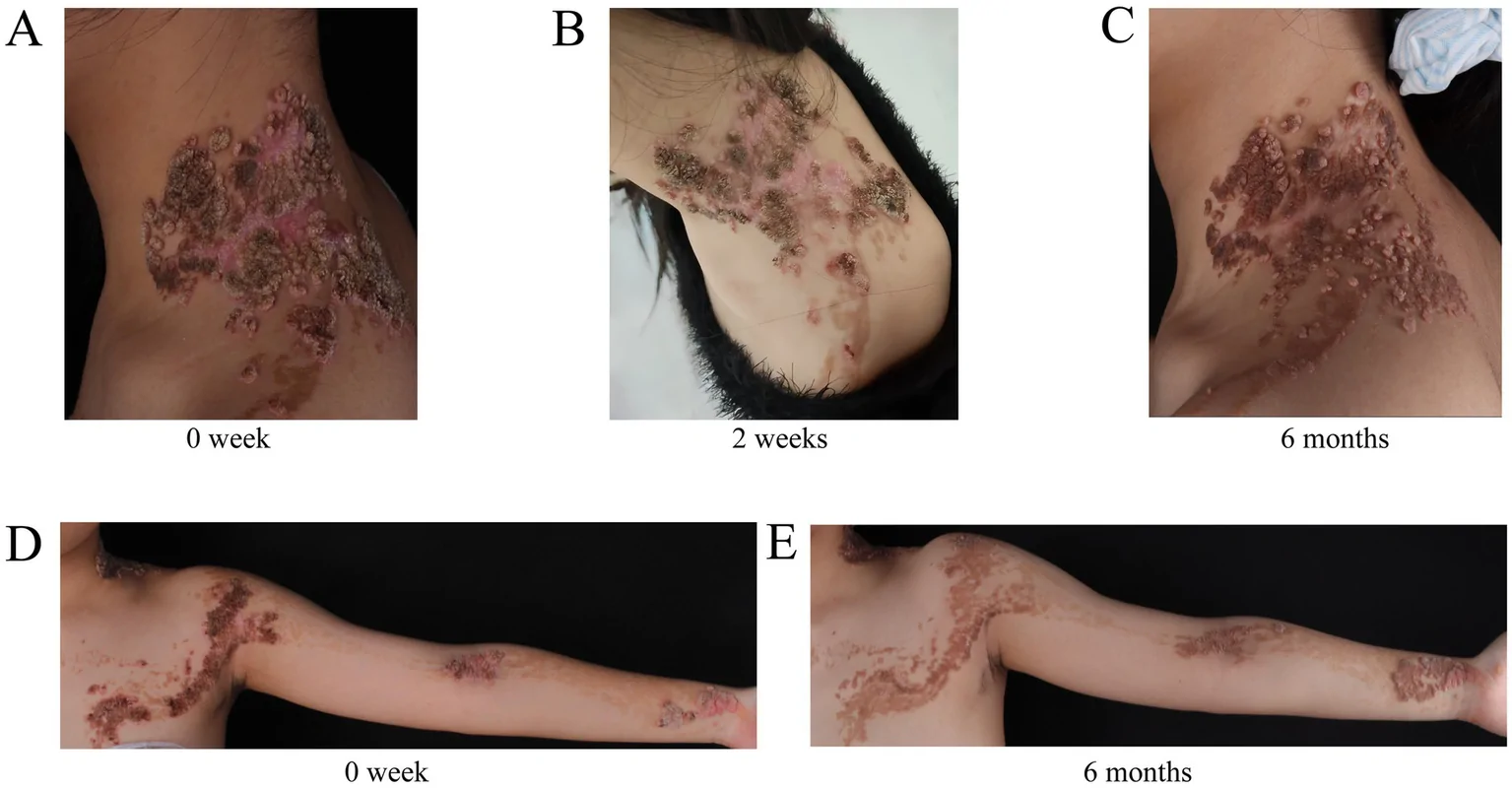
Response to topical sirolimus treatment. **(A)** Lesion on the left neck before treatment; **(B)** Lesion on the left neck after 2 weeks of treatment. **(C)** Lesion on the left neck after 6 months of treatment. **(D)** Lesion on the left upper limb before treatment; **(E)** Lesion on the left upper limb after 6 months of treatment.

**Figure 2:**
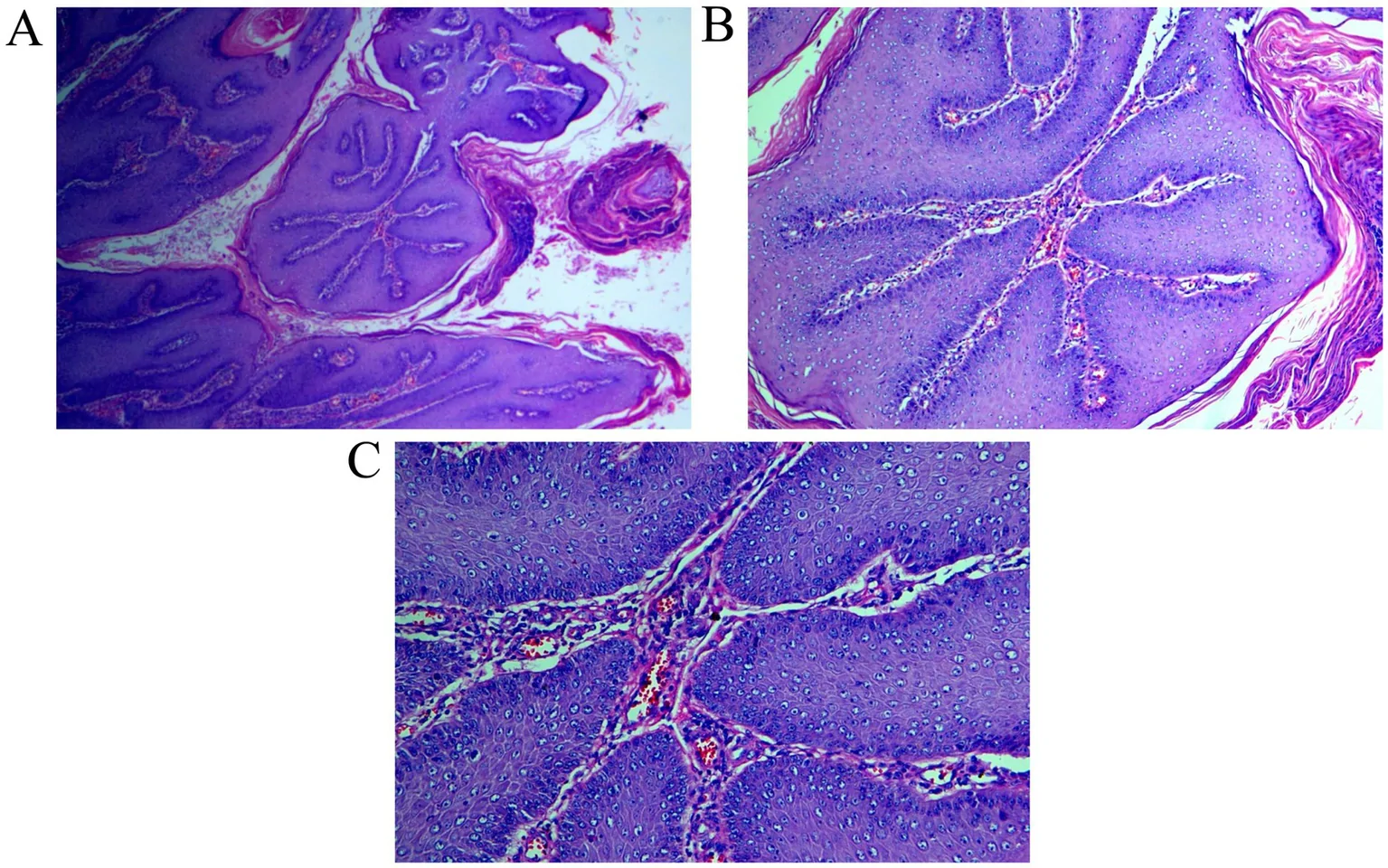
Histopathological findings of the lesion on the left neck. Hyperkeratosis of the epidermis with focal parakeratosis, thickening and hyperplasia of the spinous layer, papillomatous hyperplasia, increased pigmentation in the basal layer, and a sparse lymphocytic and histiocytic infiltrate in the superficial dermis. **(A)** HE × 40; **(B)** HE × 100; **(C)** HE × 400.

A 13-year-old child presented with light brown linear macules distributed along Blaschko’s lines since birth. The lesions progressively enlarged, thickened, and showed increased pigmentation with age. No treatment was received during this period. Recently, the child experienced self-perceived acceleration of lesion growth, prompting the visit to our hospital. Physical examination at our hospital revealed lesions distributed along Blaschko’s lines on the left neck and left upper extremity, presenting as continuous brown verrucous hyperplastic plaques with papillary projections and a rough surface, accompanied by scattered miliary light brown papules at the margins (Figures 1A,D). Histopathological examination showed hyperkeratosis with focal parakeratosis, acanthosis, papillomatous hyperplasia, increased pigmentation in the basal layer, and sparse lymphocytic and histiocytic infiltration in the superficial dermis (Figures 2A–C). Based on the above clinical features, differential diagnoses include lichen striatus, linear lichen planus, and linear psoriasis. Lichen striatus is characterized histopathologically by a band-like lymphocytic infiltrate in the superficial dermis without significant acanthosis; linear lichen planus shows interface dermatitis and wedge-shaped hypergranulosis; and linear psoriasis is often associated with Munro microabscesses. In our patient, the onset at birth, the typical verrucous lesions, and the histopathological findings of hyperkeratosis, acanthosis, and papillomatous hyperplasia, without the aforementioned specific changes, are consistent with the diagnosis of VEN.

The child initially received surgical excision treatment, but the procedure was complicated by postoperative incision infection and dehiscence, resulting in a hypertrophic scar. About 1 month after the surgery, new warty hyperplasia appeared in the original lesion area and adjacent areas of the child. Due to the unsatisfactory surgical outcome, the child subsequently tried topical retinoic acid cream and oral acitretin, but no significant improvement was achieved. Due to the continuous progression of the child’s skin lesions and poor response to conventional treatment, whole exome sequencing was performed, which identified a c.38G>T (p.Gly13Val) mosaic mutation in the *HRAS* gene, with a variant allele frequency (VAF) of 23.6%. This variant is a rare *de novo* variant and is not documented in gnomAD database.

Based on the results of genetic testing and after reviewing the relevant literature (6, 7), we initiated treatment with topical 0.5% (w/w) sirolimus ointment, a commercially available product, applied to the lesion on the left neck of the child twice daily, in the morning and in the evening, with each application of approximately 0.2 g over an area of about 70 cm^2^. After 2 weeks of treatment, the brown verrucous plaque on the left neck showed slight thinning with partial crusting and desquamation, and the pigmentation slightly improved. (Figure 1B). Given the initial response, we continued treatment with topical 0.5% (w/w) sirolimus ointment for the generalized skin lesions, still twice daily, in the morning and in the evening, with each application of approximately 0.3 g over an area of about 130 cm^2^. During the treatment period, safety was assessed at regular follow-up visits by monitoring for local adverse reactions such as skin irritation and secondary infection, as well as any systemic symptoms. At 6 months of treatment, the generalized lesion area showed a reduction, with concurrent thinning, improved pigmentation, decreased roughness, and no new lesions (Figures 1C,E), as evaluated independently by two dermatologists who reached consensus. The child’s mother reported that the topical regimen was easy to apply, well tolerated, with acceptable treatment burden, and that the improvement in lesion appearance alleviated the child’s psychological and social burden. During the 6-month treatment period, no local or systemic adverse reactions were observed (Table 1).

**Table 1:** Key clinical course and treatment timeline.

Time point	Event	Outcome
Birth	Linear macules along Blaschko’s lines on left neck/upper limb	Lesions gradually enlarged, thickened, and showed increased pigmentation with age
Age 13 (presentation)	Presented with accelerated growth; surgical excision	Postoperative infection and dehiscence with hypertrophic scarring, and new lesions at original site and adjacent areas
1–2 months post-surgery	Topical retinoic acid cream and oral acitretin	No improvement; lesions progressed
3 months post-surgery	Whole exome sequencing	*HRAS* c.38G>T (p.Gly13Val) mosaic mutation detected
Week 2 of treatment	0.5% (w/w) sirolimus ointment on left neck lesion	Left neck lesion: slight thinning and improved pigmentation
From week 2 to month 6 of treatment	Treatment extended to generalized lesions	Generalized lesions: area reduction, thinning, improved pigmentation, and decreased roughness; no local or systemic adverse reactions observed

## Discussion

VEN is a benign skin disorder caused by abnormal proliferation of epidermal cells. Its typical feature is well-defined, pigmented or dark-brown, raised skin lesions that can affect various parts of the body, often presenting in patches or along Blaschko’s lines (8). At present, there are various treatment options available for VEN in clinical practice, including topical medications, cryotherapy, laser treatment, and surgical excision. However, all these methods have limitations, such as inconsistent efficacy, potential recurrence, possible scarring, or functional impairment (4). Based on the limitations of existing treatments, recent case reports and systematic studies have proposed new approaches. For instance, the EGFR inhibitor erlotinib has shown significant therapeutic effects in extensive inflammatory linear verrucous epidermal nevus caused by acquired mutations in the TRPM4 gene (6). Another study found that EGFR inhibitors also have outstanding efficacy for EGFR mutation-related syndromes, while MEK inhibitors have limited efficacy. This underscores the importance of genetic testing in the selection of treatment plans (9). These research findings consistently indicate that precise targeted treatments targeting specific gene mutations are expected to become a key breakthrough direction in the future treatment of VEN.

Mammalian target of rapamycin (mTOR) is a key serine/threonine protein kinase in the PI3K-related kinase (PIKK) family and occupies a central position in the PI3K/AKT/mTOR signaling pathway. Its functions include integrating growth factor and energy signals and regulating cell proliferation and differentiation processes (10). Dysregulation of this signaling pathway can lead to various skin diseases such as psoriasis, pemphigus, and melanoma (11). Studies have shown that the pathogenesis of VEN is associated with mutations in multiple genes, including *HRAS* (12), *FGFR3* (13), *K10* (14), and *GJA1* (15). A small-scale study has revealed that approximately 40% of patients with VEN have mosaic mutations in the *RAS* gene, with *HRAS* mutations being the most common (12). *RAS* mutations can promote GTP binding or inhibit GTP hydrolysis, causing RAS proteins to escape upstream inhibition and continuously activate downstream signaling pathways, especially the PI3K/AKT/mTOR signaling pathway. Activation of this pathway drives excessive cell proliferation and aberrant differentiation (16, 17), which may consequently contribute to the development of VEN. *HRAS* mosaic mutations are not only a driving factor for VEN but are also closely related to the occurrence of various malignant tumors such as rhabdomyosarcoma and urothelial carcinoma (18).

Sirolimus is a highly effective and specific mTOR inhibitor that directly inhibits mTOR kinase activity and interferes with downstream signal transduction, thereby suppressing excessive cell activation and abnormal proliferation (19). Due to the favorable efficacy and safety profile of its topical formulations, 0.2% (w/w) sirolimus gel has become the first topical medication approved by the FDA for treating facial angiofibromas in TSC patients aged 6 years and older (20). Notably, this topical preparation has also shown potential efficacy in the treatment of sebaceous nevus, epidermal nevus (7), and TSC-related hypopigmented macules and subungual fibromas (21), providing clues for its application in other proliferative skin lesions.

Although there are currently no systematic reports on the use of topical sirolimus for VEN, this case, together with relevant literature, suggests that the drug may offer a new treatment option for VEN patients who do not respond to conventional therapies. This child received multiple conventional treatments, including surgical excision, topical tretinoin cream, and oral acitretin, but none of which achieved satisfactory results. However, following topical treatment with 0.5% (w/w) sirolimus ointment, the left neck lesion showed regression and thinning within 2 weeks, and at 6 months the lesion area had decreased, with further thinning, improved pigmentation, and no new lesions elsewhere. This therapeutic response suggests that the mTOR pathway may be involved in the pathogenesis of *HRAS* mosaic mutation-related VEN, and provides preliminary clinical evidence for targeted therapy of this disease.

This case report has several notable strengths, including molecular characterization and histopathological confirmation, targeted treatment based on biological plausibility, and longitudinal photographic documentation. Nevertheless, several limitations should be considered when interpreting these findings, including the single-patient design, absence of a comparator, potentially subjective assessment of response, limited duration of follow-up, and lack of pharmacokinetic or systemic exposure assessment.

## Conclusion

VEN remains a major clinical challenge, not only showing poor response to conventional treatment but also having a high recurrence rate. This case suggests that early genetic testing may be helpful in guiding individualized targeted treatment for patients with VEN who have a poor response to conventional treatment. In this case, the child was diagnosed with a mosaic *HRAS* mutation through genetic testing and received topical sirolimus treatment, achieving preliminary efficacy. This suggests that this treatment may represent a novel therapeutic approach for VEN and other skin disorders associated with mosaic *HRAS* mutations. However, recent clinical evidence regarding the use of topical sirolimus for VEN is still limited, and further research is needed to evaluate its long-term efficacy and safety.

## Data Availability

The raw data supporting the conclusions of this article will be made available by the authors, without undue reservation.
